# Tachycardia and Pre-existing Chronic Kidney Disease Are Predictors of the Worse Clinical Outcomes in Patients Recently Hospitalized With Acute Heart Failure

**DOI:** 10.7759/cureus.15802

**Published:** 2021-06-21

**Authors:** Leonardo P Suciadi, Kevin Wibawa, Giovanni Jessica, Joshua Henrina, Irvan Cahyadi, Bryany T Santi, Titus K Hariadi, Firman Tedjasukmana, Nathania M Kristanti, Elisa F Pakpahan, Reynold A Manullang, Antono Sutandar

**Affiliations:** 1 Cardiology, Siloam Heart Institute/Siloam Hospitals Kebon Jeruk, Jakarta, IDN; 2 Research, Siloam Heart Institute, Jakarta, IDN; 3 Epidemiology, School of Medicine and Health Sciences Atma Jaya Catholic University of Indonesia, Jakarta, IDN

**Keywords:** heart failure, mortality, rehospitalization, tachycardia, chronic kidney disease

## Abstract

Background: This study aimed to assess the factors contributing to the outcomes of recently hospitalized patients with heart failure (HF).

Methods: A prospective data of 76 adults who were admitted due to acute HF between October 1, 2019 and June 30, 2020 at our center were analyzed. Endpoints included survival and rehospitalization within six months after discharge.

Results: The mean age was 64.9 ± 13.8 years, with a male preponderance (68.4%). Approximately 60.5% of patients had the left ventricular ejection fraction (LVEF) <40%, whereas 26.3% of patients had LVEF ≥50%. Coronary artery disease (75%), arterial hypertension (72.4%), chronic kidney disease (46.1%), and diabetes mellitus (46.1%) were the most frequent comorbidities. Poor compliance (40.8%) and non-cardiac infection (21.1%) were the common precipitating factors for hospitalization. The majority of subjects had severe symptoms, indicated by the frequent need of intensive care unit (43%), high N-terminal prohormone brain natriuretic peptide levels [NT-proBNP; median, 4765 (1539.7-11782.2) pg/mL], and presence of either atrial fibrillation, severe mitral regurgitation, or significant pulmonary hypertension in approximately one-third of cases. Even though in-hospital mortality was relatively low (2.6%), the all-cause mortality and rehospitalization rates in the next six months after discharge were still high, reaching 22.54% and 19.72%, respectively. Further survival analysis showed that tachycardia on admission and pre-existing chronic kidney disease (CKD) resulted in low six-month survival rates among these patients.

Conclusion: After hospital discharge, patients with HF were still exposed to higher risks of death and readmission albeit with the medication addressed. Tachycardia on admission and pre-existing CKD might predict worse outcomes.

## Introduction

Heart failure (HF) is a major global problem and the leading cause of morbidity and mortality worldwide [1]. HF is the primary reason for hospitalization in the elderly population, and the number of readmissions is high in this population at risk [2]. These data showed that HF is a crucial issue and challenge for health services in the community. Acute HF (AHF) refers to the rapid onset or worsening of symptoms and/or signs of HF, which require prompt treatment and frequently related to unplanned hospital admission or emergency room visit [3]. Generally, AHF encompasses a complex syndrome, including various clinical presentations, underlying cardiac problems, trigger factors, comorbidities, and complications, leading to a relatively high mortality rate during hospitalization and after discharge [4]. Therefore, patient-based, rather than disease-based, data might provide essential information to evaluate miscellaneous aspects of hospitalized patients with AHF [5,6]. Consequently, this approach could improve the clinical assessment and management strategy to reduce in-hospital mortality and rehospitalization rates caused by AHF.

To date, there are numerous published data on the epidemiology, clinical management, and outcomes of AHF in Western countries. However, similar information is still lacking and less comprehensive in Southeast Asian countries. Multiple factors, including different cultures, less organized systems, limited access to healthcare, poor adherence of patients, limited diagnostic or therapeutic tools, and lack of trained investigators, affect the scarce data provided in most Asian countries, including Indonesia [6,7]. Moreover, this lack of data is a major barrier to participation in global collaboration of the relevant issue [8]. Notably, several published registries of AHF in Asian countries showed that the demographic characteristics, comorbidities, treatment approaches, and outcomes of patients with AHF are slightly different from the Western data [9,10]. Thus, it is pivotal to identify any differences to elaborate on whether the Western data and guidelines regarding the management of patients with AHF can be practically applied to Asian patients, especially in Indonesia.

This study aimed to describe the clinical characteristics and analyze the essential factors contributing to outcomes of hospitalized patients with AHF. This study will be of value in adding databases of AHF during hospitalization and until six months after discharge, especially in the Indonesian population. Thus, the results might lead to a protocol to reduce mortality and rehospitalization rates related to AHF.

## Materials and methods

This prospective study included all patients aged ≥18 years who were admitted to our hospital with the primary indication of AHF between October 1, 2019 and June 30, 2020, at Siloam Hospital Kebon Jeruk, Jakarta. AHF was defined based on the criteria delineated in 2016 European Society of Cardiology guidelines for HF [3]. Patients were further categorized to acute de novo HF (admitted for the first time without a history of HF) and acute decompensated HF (ADHF; sudden worsening of HF in previously diagnosed or hospitalized patients). Patients who were discharged or transferred by their own intention before fulfilling treatment or admitted by other primary reasons than AHF were excluded.

Data regarding the patients’ demographic, clinical characteristics, comorbidities, history of cardiovascular procedures, HF etiology, precipitating factors, laboratory investigation, electrocardiography, echocardiography, and management during hospitalization were directly collected from hospital medical records. The outcomes were in-hospital mortality, defined as all-cause death during hospitalization for AHF; six-month rehospitalization, defined as rehospitalization for HF within six months after hospital discharge; and six-month mortality, defined as all-cause mortality within six months after hospital discharge. The outcome data regarding in-hospital mortality were collected from the medical records, and data on six months of rehospitalization and mortality were collected by either telephone contact or clinical visit within six months after hospital discharge.

Descriptive statistics were used to summarize the data. Categorical variables were presented as frequencies and percentages. For continuous variables, Shapiro-Wilk or Kolmogorov-Smirnov normality test was performed as appropriate. Normally distributed data were summarized as mean and standard deviation. Otherwise, median and interquartile range (25th and 75th percentiles) were used. Chi-square test or Fischer’s exact test were performed to compare and determine the independent variables according to six-month mortality that will be included in the logistic regression model. Independent variables included in the Cox regression model were variables with chi-square or Fisher’s exact ρ-value < 0.25. Survival estimates for six-month mortality were calculated using the Kaplan and Meier method. Statistical analysis was conducted using SPSS version 22.0.

## Results

This study included a total of 76 patients admitted with the primary complaint of AHF during a nine-month registry period. The characteristics, comorbidities, and history of cardiovascular procedures are presented in Table 1. The mean age of the subjects was 64.9 ± 13.8 years, and the majority of patients (68.4%) were male. Notably, nearly one-third of our patients were very elderly with age ≥75 years. The median body mass index of the patients was 24.6 (22.6-27.3) kg/m^2^. Moreover, 71% of subjects were classified as overweight or obese, and only 3.9% of them were underweight (BMI < 18.5 kg/m^2^).

**Table 1:** Basic characteristic of the study cohort. BMI, body mass index; CABG, coronary artery bypass graft; CAD, coronary artery disease; CKD, chronic kidney disease; COPD, chronic obstructive pulmonary disease; CRT, cardiac resynchronization therapy; DM, diabetes mellitus; ICCU, intensive cardiac care unit; IQR, interquartile range; PCI, percutaneous coronary intervention; SD, standard deviation; TIA, transient ischemic attack.

Characteristics	n (%)
Age, mean ± SD (years)	64.96 ± 13.79
≥75 years	22 (28.95)
<75 years	54 (71.05)
Male sex	52 (68.4)
BMI, kg/m^2^, median (IQR)	24.65 (22.6–27.34)
Underweight	3 (3.95)
Normal	19 (25)
Overweight	19 (25)
Obese	35 (46.05)
Duration of hospitalization in the cardiac ward, median (IQR), days	5 (4–9)
Duration of hospitalization in the ICCU, median (IQR), days	4 (2.5–7)
Number of patients needing intensive cardiac care	33 (43.4)
Smoking habit	
Active smoker	12 (15.8)
Ex-smoker	8 (10.5)
Never smoke	56 (73.7)
Comorbidity	
Patients with cardiovascular comorbidity	4 (5.3)
Patients with non-cardiovascular comorbidity	8 (10.5)
Patients with both cardiovascular and non-cardiovascular comorbidities	63 (82.9)
CAD	57 (75)
Hypertension	55 (72.4)
CKD without hemodialysis	35 (46.1)
Non-insulin-dependent DM	34 (46.1)
Obesity	33 (43.4)
Other comorbidities	25 (32.9)
Persistent/permanent atrial fibrillation	19 (25)
Other systemic diseases (autoimmune, chronic inflammatory, connective tissue)	11 (14.5)
Insulin-dependent DM	7 (9.2)
Geriatry and frailty	6 (7.9)
Stroke/TIA	6 (7.9)
Chronic liver disease	4 (5.3)
CKD on hemodialysis	3 (3.9)
COPD	3 (3.9)
Chronic anemia	2 (2.6)
Cachexia	1 (1.3)
Cancer	1 (1.3)
History of cardiovascular procedure	
PCI	14 (18.4)
CABG	11 (14.5)
Heart valve repair/replacement	4 (5.3)
Other cardiovascular procedures (angiography)	3 (3.9)
CRT	1 (1.3)

Approximately 43% of patients required intensive care during hospitalization. Meanwhile, the duration of hospitalization in the cardiac ward and intensive care unit was 5 (4-9) days and 4 (2.5-7) days, respectively. The common comorbidities observed among our patients were coronary artery disease (CAD) (75%), arterial hypertension (72.4%), chronic kidney disease (CKD) without hemodialysis (46.1%), and non-insulin-dependent diabetes mellitus (46.1%). Some patients had a history of undergoing previous cardiovascular procedures including percutaneous coronary intervention (18.4%), coronary artery bypass grafting (14.5%), surgical valve repair/replacement (5.3%), and cardiac resynchronization therapy (1.3%).

Most patients (65.8%) had systolic blood pressure between 90 and 139 mmHg at the time of admission, whereas the median heart rate was 88 beats per minute (bpm) (Table 2). Underlying CAD was the most common etiology for AHF, encompassing chronic coronary syndrome (50% cases), prior myocardial infarction (17.1%), and acute coronary syndrome (15.8%). Based on the time interval of the event, only 13.2% of patients had de novo HF, whereas the remaining patients had ADHF. Meanwhile, with respect to the clinical profiles, the majority of patients (88.2%) presented with ADHF, and only 10.5% and 1.3% of them had acute lung edema with respiratory distress and cardiogenic shock, respectively. In patients with ADHF, the common precipitating factors were poor compliance (40.8%), non-cardiac infection (21.1%), acute coronary syndrome (9.2%), hypertensive emergency (3.9%), and acute mechanical cause (2.6%).

**Table 2:** Clinical presentation, etiology, onset, precipitating factor, and AHF clinical profile of study cohort. ACS, acute coronary syndrome; ADHF, acute decompensated heart failure; AHF, acute heart failure; bpm, beats per minute; DBP, diastolic blood pressure; DCM, dilated cardiomyopathy; HF, heart failure; HR, heart rate; IQR, interquartile range; JVP, jugular venous pressure; SBP, systolic blood pressure; SD, standard deviation; VHD, valvular heart disease.

Variables	n (%)
Clinical presentation	
SBP at admission, mmHg, median (IQR)	127 (105–144)
SBP at admission < 90 mmHg	4 (5.3)
SBP at admission 90–139 mmHg	50 (65.8)
SBP at admission ≥ 140 mmHg	21 (28.9)
DBP at admission, mmHg, mean ± SD	77 ± 21
HR at admission, bpm, median (IQR)	88 (77–105)
Raised JVP	23 (30.3)
Lung crepitations	65 (85.5)
Leg edema	39 (50.3)
Ascites	7 (9.2)
Pleural effusion	17 (22.4)
Cyanosis	1 (1.3)
Etiology	
Chronic coronary syndrome	38 (50)
Old myocardial infarction with scar tissue	13 (17.1)
VHD	13 (17.1)
Mitral stenosis	1 (1.3)
Mitral regurgitation	5 (6.6)
Aortic stenosis	4 (5.3)
Aortic regurgitation	2 (2.6)
Etiology of VHD	
Infective endocarditis	1 (1.3)
Degenerative	2 (2.6)
Rheumatic heart disease	1 (1.3)
Congenital bicuspid/prolapse	2 (2.6)
Other etiologies of VHD	5 (6.6)
ACS	12 (15.8)
Hypertensive heart disease	12 (15.8)
Idiopathic/familial DCM	4 (5.3)
Metabolic/nutritional	2 (2.6)
Other etiologies	1 (1.3)
AHF onset	
De novo	10 (13.2)
ADHF from chronic HF	66 (86.8)
AHF precipitating factor	
Poor compliance	31 (40.8)
Non-cardiac infection	16 (21.1)
ACS	7 (9.2)
Hypertensive emergency	3 (3.9)
Acute mechanical cause	2 (2.6)
Others (high blood sugar, suboptimal drug dose)	11 (14.5)
AHF clinical profile	
ADHF	67 (88.2)
Acute lung edema (respiratory distress)	8 (10.5)
Cardiogenic shock	1 (1.3)

As shown in Table 3, the mean hemoglobin level at admission was 12.51 ± 2.3 mg/dL. Most patients had renal impairment with a median creatinine level of 1.7 (1.2-2.6) mg/dL, indicating a median estimated glomerular filtration rate of 38 (22.9-59.6) mL/min. Unfortunately, only a few patients (31.6%) underwent N-terminal prohormone brain natriuretic peptide (NT-proBNP) tests, and the median level of this cardiac biomarker at the time of admission was 4765 (1539.7-11782.2) pg/mL. Atrial fibrillation was identified in 29% of initial electrocardiography (ECG) of our patients. Widened QRS complex ≥120 ms on ECG was found in 13.3% of subjects. The premature ventricular complex was common in the ECG result, which was noted in 13.3% of patients.

**Table 3:** Laboratory, electrocardiographic, and echocardiographic results of the study cohort. bpm, beats per minute; Cr, creatinine; ECG, electrocardiography; eGFR, estimated glomerular filtration rate; HR, heart rate; IQR, interquartile range; LA, left atrium; LAVi, left atrial volume index; LV, left ventricle; LVEF, left ventricular ejection fraction; NT-proBNP, N-terminal prohormone brain natriuretic peptide; PASP, pulmonary artery systolic pressure; PVC, premature ventricular complex; RDW, red cell distribution width; RV, right ventricle; SD, standard deviation; SGOT, serum glutamic oxaloacetic transaminase; SGPT, serum glutamic pyruvate transaminase; TAPSE, tricuspid annular plane systolic excursion; TIBC, total iron-binding capacity; TSAT, transferrin saturation.

Variable	Value
Laboratory results, median (IQR), unless specified otherwise	
Hemoglobin, mean ± SD, g/dL	12.51 ± 2.3
RDW, mean ± SD, % (n = 3)	16.7 ± 4.91
Serum iron, mean ± SD, µg/dL (n = 14)	62.71 ± 54.72
TIBC, µg/dL, (n = 14)	201 (193.25–238.25)
TSAT, mean ± SD, % (n = 14)	40.26 ± 26.52
Ferritin, ng/mL, (n = 14)	279.11 (126.4–526.14)
Cr, mg/dL	1.7 (1.2–2.58)
eGFR, mL/min	38.06 (22.94–59.64)
Sodium, mEq/L	138 (135–140)
Potassium, mEq/L	3.9 (3.43–4.5)
Total bilirubin, mg/dL (n = 17)	1.63 (0.99–5.05)
Albumin, mean ± SD, mg/dL (n = 21)	3.24 ± 0.66
NT-proBNP, pg/mL (n = 24)	4765 (1539.75–11782.25)
Troponin T	
Troponin T-test, n (%)	22 (28.9 %)
Increased value of troponin T-test, n (%)	13 (59.1%)
SGOT, U/L (n = 59)	29 (19–49)
SGPT, U/L (n = 59)	22 (16–35)
ECG, n (%), unless specified otherwise	
Rhythm	
Sinus	51 (67.1)
Atrial fibrillation	22 (29)
Paced	1 (1.3)
Other rhythms	2 (2.6)
HR, bpm, median (IQR) (n = 75)	94 (79–119)
QRS duration, ms, median (IQR) (n = 75)	80 (80–100)
QRS duration < 120 ms	65 (86.7)
QRS duration ≥ 120 ms	10 (13.3)
QTc duration, msec, median (IQR) (n = 75)	437 (400–460)
QTc duration ≤ 460 ms	58 (77.3)
QTc duration > 460 ms	17 (22.7)
PVC (n = 75)	
Without	65 (86.7)
Infrequent	6 (8)
Frequent	4 (5.3)
Echocardiography, n (%) unless specified otherwise	
LA diameter (n = 50)	
≥40 mm	40 (80.0)
<40 mm	10 (20.0)
LAVi, mL/m^2^, mean ± SD (n = 11)	58.91 ± 24.36
LV EDD, mm, mean ± SD (n = 36)	55.13 ± 8.953
E/e’, median (IQR) (n = 37)	19.00 (12.75–23.00)
LVEF	
≥50%	20 (26.3)
40–49%	10 (13.2)
<40%	46 (60.5)
Diastolic dysfunction (n = 53)	
Present	48 (90.57)
No diastolic dysfunction	-
Unable to evaluate	5 (9.43)
Grading of diastolic dysfunction (n = 48)	
Grade I	8 (16.67)
Grade II	20 (41.67)
Grade III	20 (41.67)
Mitral regurgitation (n = 59)	
Severe	16 (27.12)
Mild––moderate	26 (44.07)
No mitral regurgitation	17 (28.82)
PASP (n = 46)	
≥50 mmHg (high likelihood)	18 (39.13)
35–50 mmHg (intermediate likelihood)	19 (41.30)
<35 mmHg (low likelihood)	9 (19.56)
TAPSE (n = 47)	
<17 mm	19 (40.42)
≥17 mm	28 (59.58)
RV base size (n = 45)	
≥42 mm	14 (31.11)
<42 mm	31 (68.89)

By echocardiography study, the left ventricular ejection fraction (LVEF) < 40% was found in 60.5% of patients, whereas LVEF ≥50% in 26.3% of subjects. The LVEF was measured using the standard of Simpson’s procedure from the apical four-chamber view. Since most patients had diastolic dysfunction, restrictive diastolic dysfunction was noted in 41.7% of patients. Nearly three-quarters of patients had mitral regurgitation, and severe regurgitation was identified in 27.1% of cases. More than one-third of subjects had estimated pulmonary artery systolic pressures >50 mmHg, indicating a high likelihood of pulmonary hypertension. The right ventricular dysfunction as simply indicated by reduced tricuspid annular plane systolic excursion was found in 40.4% of subjects.

During hospitalization, serious ventricular arrhythmias (ventricular tachycardia/fibrillation) occurred in 6.6% of patients. However, electrical cardioversion was only needed in one patient with unstable hemodynamics during prolonged ventricular tachycardia, as shown in Table 4. Some corresponding procedures required included noninvasive positive pressure ventilation (9.2%), invasive mechanical ventilation (6.6%), hemodialysis or ultrafiltration (6.6%), and insertion of the intra-aortic balloon counterpulsation (2.6%).

**Table 4:** In-hospital management of the study cohort. bpm, beat per minute; BiPAP, bi-level positive airway pressure; CABG, coronary artery bypass graft; CPAP, continuous positive airway pressure; CPR, cardiopulmonary resuscitation; CRT, cardiac resynchronization therapy; IABP, intra-aortic balloon pump; ICD, implantable cardioverter-defibrillator; SD, standard deviation; TdP, torsades des pointes; VF, ventricular fibrillation; VT, ventricular tachycardia.

Variables	N (%)
Complication during admission	
Respiratory distress and respiratory failure	-
Cardiogenic shock	-
Ventricular tachyarrhythmia (VT, VF, TdP)	5 (6.6)
Atrial fibrillation > 150 bpm	-
Cardiac arrest	-
Non-cardiac complication	5 (6.6)
Injection drugs administered	
Furosemide or other diuretics	70 (92.1)
Nitrate	33 (43.4)
Dobutamine	12 (15.8)
Dopamine	2 (2.6)
Norepinephrine	8 (10.5)
Milrinone	-
Anticoagulant	16 (21.1)
Blood product	
Whole blood	-
Packed red cells	7 (9.2)
Volume, mL, mean ± SD	537.67 ± 364
Fresh frozen plasma, mean ± SD volume, mL	3 (3.9)
Volume, mL, mean ± SD	834 ± 364
Amiodarone	4 (5.3)
Digoxin	7 (9.2)
Lidocaine	3 (3.9)
Antibiotic	25 (32.9)
Iron supplementation	1 (1.3)
Cardiac procedures, during admission	
Percutaneous coronary intervention	4 (5.3)
Percutaneous structural intervention	1 (1.3)
CABG	3 (3.9)
Open valve surgery	2 (2.6)
IABP	2 (2.6)
CRT implantation	-
ICD implantation	-
Electrical cardioversion/defibrillation shock	1 (1.3)
Intubation and mechanical ventilation	5 (6.6)
Duration of mechanical ventilation, days, mean ± SD	4.83 ± 4.4
BiPAP/CPAP	7 (9.2)
Hemodialysis/ultrafiltration	5 (6.6)
CPR	-
Others	12 (15.8)

Regarding the management of patients, loop diuretics and nitrates were the most common intravenous drugs used, which contributed to 92.1% and 43.4% of patients, respectively. Additionally, inotropes were needed in approximately one-third of the subjects, with dobutamine and norepinephrine as the most preferred agents. Only 21.1% of patients received parenteral anticoagulation for venous thromboembolism treatment or prophylaxis.

Importantly, the negative fluid balance was achieved in almost all patients (93%) during hospital stay (Table 5). Table 6 showed the common medications prescribed at discharge included diuretics (77.6%), spironolactone (59.2%), angiotensin-converting enzyme (ACE) inhibitors/angiotensin receptor blocker (ARB) (56.6%), and beta-blockers (44.7%). Meanwhile, other familiar HF drugs, such as digoxin, sacubitril/valsartan, ivabradine, and SGLT2 inhibitors were only prescribed in ≤ 10%.

**Table 5:** Predischarge condition of the study cohort. eGFR, estimated glomerular filtration rate; HR, heart rate; IQR, interquartile range; SD, standard deviation; SGOT, serum glutamic oxaloacetic.

Variables	Median (IQR)
SBP, mmHg (n = 74)	114 (104.5–131.0)
DBP, mmHg, median (IQR) (n = 74)	68.50 (59.0–75.25)
Heart rate, beats per minute (n = 74)	78.00 (70.00–89.25)
Body weight, kg (n = 69)	63.00 (54.15–70.00)
Body water balance, n (%) (n = 73)	
Positive	5 (6.84)
Negative	68 (93.16)
Creatinine, mg/dL	1.55 (1.10–2.40)
eGFR, mL/min	42.61 (24.33–69.94)
Sodium, mEq/L, mean ± SD	137.75 ± 3.36
Potassium, mEq/L	3.80 (3.52–4.1)
SGOT, U/L (n = 60)	29.00(19.25–52.00)
SGPT, U/L (n = 60)	22.50 (16.25–36.50)
Total bilirubin, mg/dL (n = 18)	1.83 (1.19–4.70)
Hemoglobin, g/dL	11.90 (10.55–13.87)

**Table 6:** Discharge medication prescribed for the study cohort. ACE, angiotensin-converting enzyme; NOAC, non-vitamin K oral anticoagulant.

Medication	N (%)
Furosemide	57 (75)
20 mg	7 (9.2)
40 mg	27 (35.5)
60–80 mg	14 (18.5)
> 80 mg	9 (11.8)
Hydrochlorothiazide or indapamide	2 (2.6)
Nitrate	25 (32.9)
2.5 mg	1 (1.3)
5 mg	4 (5.3)
10 mg	2 (2.6)
15 mg	6 (7.9)
>15 mg	12 (15.8)
ACE inhibitor	10 (13.2)
Angiotensin receptor blockers	33 (43.4)
Beta-blockers	34 (44.7)
Spironolactone	45 (59.2)
12.5 mg	3 (3.9)
25 mg	19 (25.0)
50 mg	14 (18.4)
100 mg	9 (11.8)
Aspirin	16 (21.1)
Clopidogrel	34 (44.7)
Ticagrelor	-
Warfarin	10 (13.2)
NOAC	6 (7.9)
Digoxin	9 (11.8)
0.125 mg	7 (9.2)
0.250 mg	2 (2.6)
Amiodarone	5 (6.6)
Ivabradine	6 (7.9)
Sacubitril/valsartan	6 (7.9)
Tolvaptan	-
Statins	50 (65.8)
Insulin	11 (14.5)
Empaglifozin/dapaglifozin	4 (5.3)
Dihydropyridine calcium channel blocker	12 (15.8)
Nondihydropyridine calcium channel blocker	3 (3.9)
Trimetazidine	2 (2.6)

**Figure 1:**
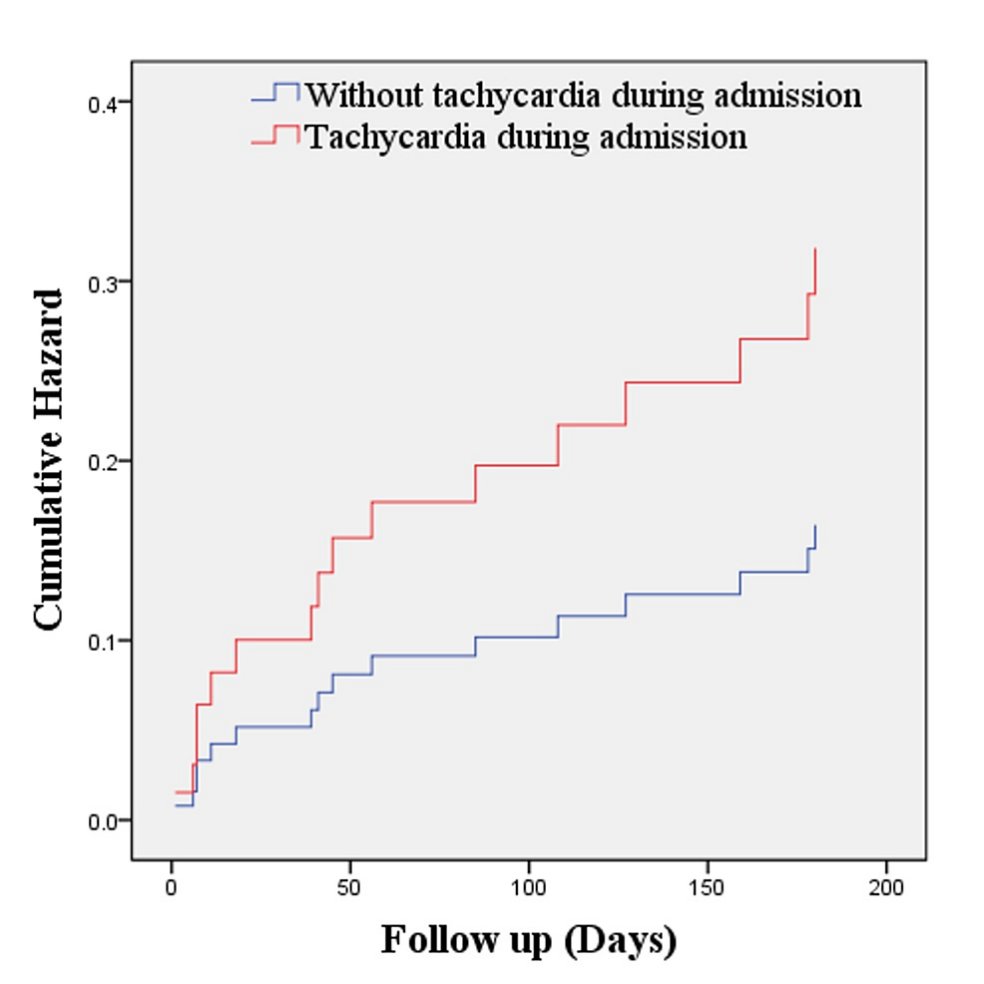
Cox regression of six-month mortality of patients with AHF with tachycardia during admission and without tachycardia during admission (p = 0.235). AHF, acute heart failure.

**Figure 2:**
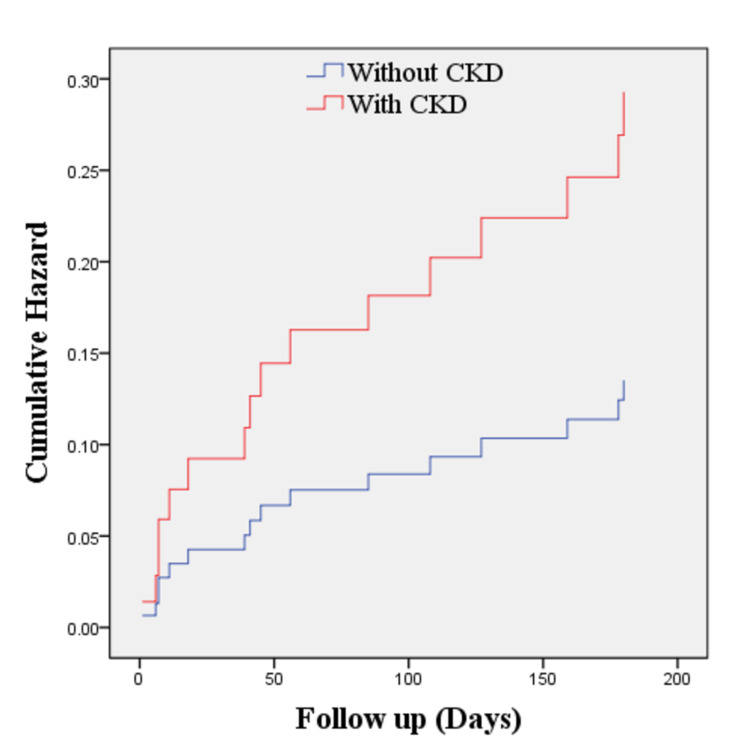
Cox regression of six-month mortality of patients with AHF with CKD and without CKD (p = 0.290). AHF, acute heart failure; CKD, chronic kidney disease.

**Figure 3:**
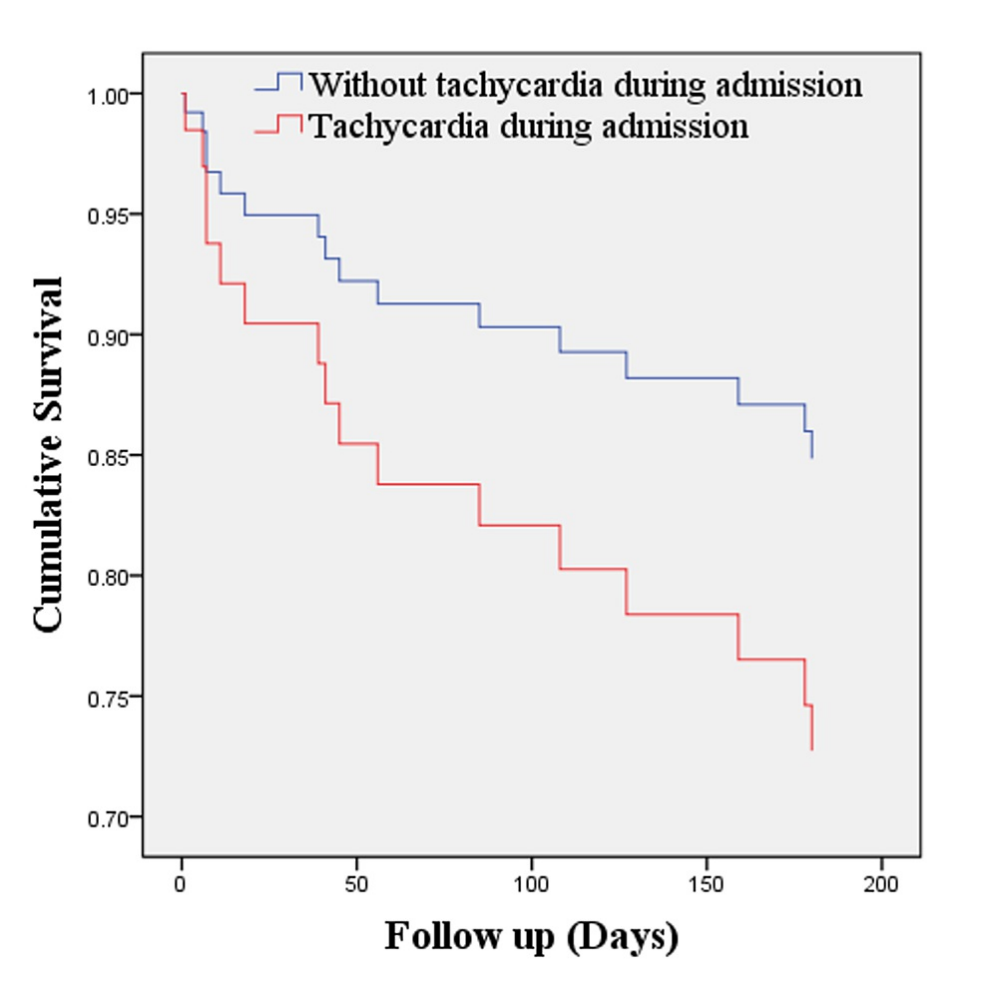
Kaplan-Meier estimate of mortality between patients with AHF with tachycardia and without tachycardia during admission (log-rank test, 0.06). AHF, acute heart failure.

**Figure 4:**
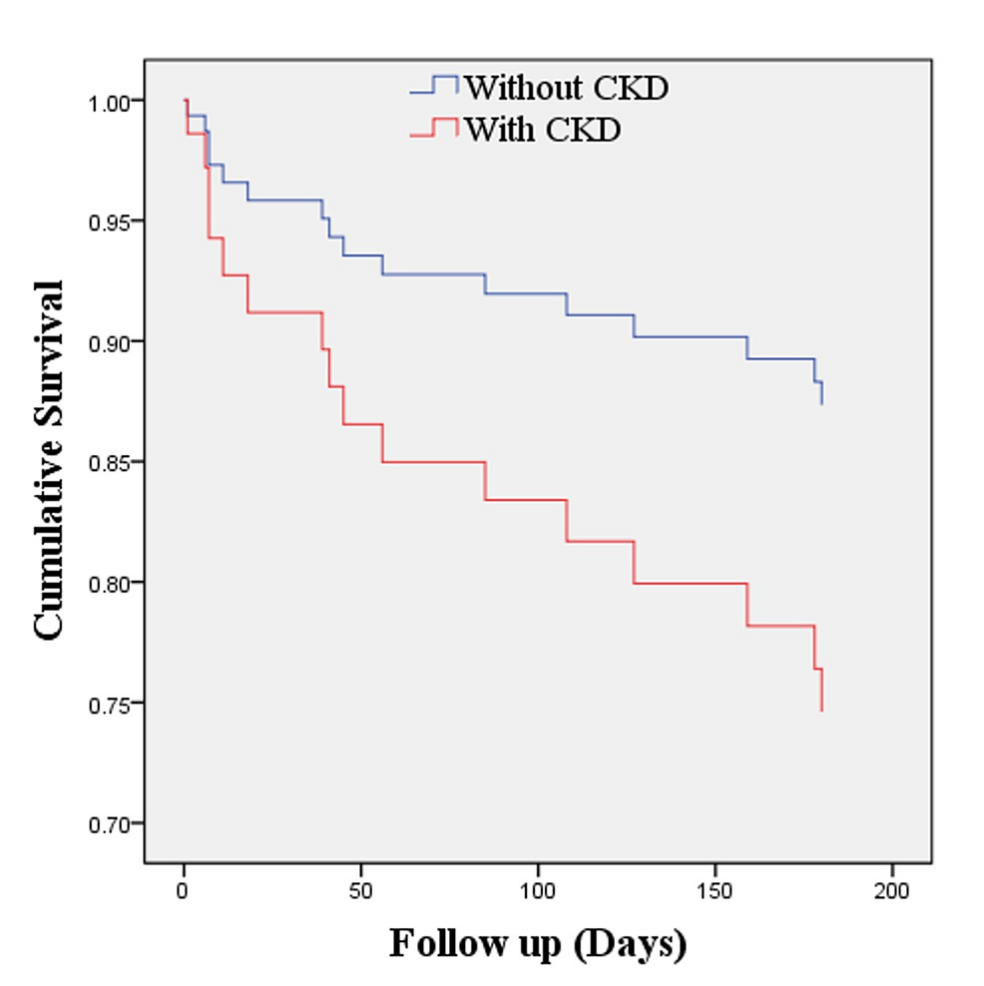
Kaplan-Meier estimate of mortality between patients with AHF with CKD and without CKD (log-rank test 0.018). AHF, acute heart failure; CKD, chronic kidney disease.

As shown in Table 7, even though in-hospital mortality was relatively low (2.6%), the number of all-cause mortality and rehospitalization in the next six months after discharge were still considerably high, reaching 22.54% and 19.72%, respectively. Cox regression analysis showed that tachycardia at admission (HR 1.98; 95% CI 0.65-5.77) and pre-existing CKD (HR 2.16; 95% CI 0.52-9.05) increased the mortality rate compared to patients without these conditions, although these findings were not statistically significant (Table 8). Further analysis of these parameters in Kaplan Meier revealed that there were poorer six months survival rates in patients with tachycardia on admission (log-rank test 0.06; Figures 1 and 2) and underlying CKD (0.018; Figures 3 and 4). Patients with initial tachycardia and previous renal dysfunction had a lower mean survival time compared to those without these conditions (133 days vs 161 days, and 136 vs 169 days, respectively). It is important to note that none of these covariates reached the median survival at six months follow-up.

**Table 7:** In-hospital mortality, six-month mortality, and six-month rehospitalization.

Variables	Total n (%)
In-hospital mortality	2 (2.6)
Six-month mortality	16 (22.54)
Six-month rehospitalization	14 (19.72)

**Table 8:** Cox regression model of six-month follow-up. CKD, chronic kidney disease; eGFR, estimated glomerular filtration rate.

Variable	HR	95% CI	P-value
Hypertension during admission	1.313	0.455–3.795	0.615
Tachycardia during admission	1.938	0.651–5.772	0.235
CKD	2.165	0.518–9.053	0.290
Anemia	1.308	0.395–4.330	0.661
eGFR <45 mL/min	1.780	0.356–8.911	0.483
Hyponatremia during admission	1.586	0.515–4.885	0.422

## Discussion

The patients in this study were younger (mean age, 64.9 years) than populations in Western studies [11], and were predominantly male and overweight or obese. Specifically, approximately one-third of patients were a very elderly group with age >75 years. CAD and hypertension were prevalent comorbidities in patients included in this registry. Interestingly, the majority of HF patients enrolled were overweight and obese. It seems that this overnutrition condition should be considered as an important comorbidity in our population. Additionally, CAD, with various clinical profiles, was the most common etiology of HF in our center and this fact is consistent with the data worldwide [9,11]. Other common causes identified in our population were hypertensive and valvular heart diseases.

At our center, acute decompensation of chronic HF was much more prevalent than the case of de novo counterpart. Importantly, poor compliance of the patients was the commonest precipitating factor of the worsening of HF symptoms, as it was found in nearly half of the admitted patients*.* This finding delineates the crucial role of patient education and sustainable services after hospital discharge to avoid rehospitalization or mortality. Patients with HF often need to undergo long-term and complex management. Thus, low health literacy may affect patient compliance to HF medication. It is understood that several patients did not understand their current situation and medication, as well as management plan of the disease. This lacking of understanding would easily result in abrupt cessation of the medication soon after the symptoms subsided [12]. Additionally, elderly patients tend to discontinue the medication when adverse drug effects appear [13]. It is important to provide adequate information regarding the common side effects of drugs to the patients. Routine evaluation of drug interaction and polypharmacy could be an effective solution to this problem.

Other frequent precipitants of ADHF in our subjects were non-cardiac infection, and pneumonia was the most common type of infection that we encountered. As generally known, patients with HF had an increased risk of developing pneumonia. Alveolar and interstitial congestion could disrupt physiological mechanisms of alveolar lining fluid as the cellular barriers. This damage would impair the microbial clearance and increase the susceptibility of the respiratory tract to infection [14]. Meanwhile, pneumonia and other systemic infections would increase cardiac workload, which is a detrimental situation for patients with chronic HF. The most common bacterial causative agents reported in patients with HF are *Streptococcus pneumoniae* and *Staphylococcus aureus* [15]. Besides, various types of viruses, such as influenza, parainfluenza virus, coronavirus, and human metapneumovirus, are also common causes of community-acquired pneumonia in this population. Nevertheless, co-infection by bacteria and viruses often occur [16,17]. Regarding this issue, the guideline recommends that patients with HF should receive pneumococcal and yearly influenza vaccination to reduce worsening of symptoms and hospitalization [3]. Besides lung infection, other non-cardiac infections, such as sepsis, urinary tract infection, and even soft tissue infection, can lead to worsening of HF symptoms and hospitalization [18].

Patients with low LVEF (<40%) dominated in this study (60.5% subjects), and this finding is similar to other Asian registries [9,11]. The higher proportion of HFrEF in our center might be correlated to CAD as the most common etiology and comorbidity encountered here. It is important to note that approximately one-third of patients with HF in this study had either atrial fibrillation, severe functional mitral regurgitation, or significant pulmonary hypertension. The high median NT-proBNP levels (4765 pg/mL) might indicate the relative severe HF symptoms in our population.

Intravenous diuretic, especially furosemide, was the most commonly administered drug during hospitalization. This agent is effective in a majority of cases of acute HF to relieve the volume overload symptoms, thus gaining negative water balance before discharge [9]. Although diuretic resistance might prohibit decongestion strategy, this problem could be solved by combining some diuretic agents [19]. Intravenous nitrates were also commonly administered to optimize symptom relief at the initial period, as long as there was no hypotension.

The in-hospital mortality rate at our center (2.6%) was considerably lower compared to the previously reported data from Indonesia, which were 6.7% and 3% [7,20]. Despite this lower death rate during hospitalization, the six-month mortality and rehospitalization rates significantly increased to 22.54% and 19.72%, respectively. Nevertheless, this six-month death rate was still lower than those of the previous reported Asian studies, which were 26.3% and 45.8% [21,22]. The relatively high mortality and hospital readmission rates within the next six months after discharge emphasized that HF is a serious disease with a rapidly progressive condition, albeit proper management during hospitalization. Thus, sustainable optimization of treatment after discharge is of paramount importance to reduce adverse events in the future. Delivering education and improving patients’ compliance might offer an effective way to obtain better long-term outcomes; particularly, poor compliance was the most prevalent trigger of rehospitalization in our center. In contrast, clinician inertia might lead to suboptimal management of patients with HF. Since the Asian population has lower body weight and higher sensitivity to drugs than the Western population, underdosing and underprescription of HF-modifying drugs were common [23]. As generally known, suboptimal doses of ACE inhibitors, ARBs, beta-blockers, and aldosterone antagonists could subsequently increase the mortality and rehospitalization rate in patients with HF, particularly HFrEF.

The Cox regression model of six-month mortality was presented in Table 8. From this study, the hazard ratios of tachycardia during admission and CKD were 1.938 and 2.165, respectively. Tachycardia on admission and CKD increase the risk of mortality at the six-month follow-up even though it is not statistically significant. It can as the effect of a smaller number of respondents compared to other studies. Assessment for tachycardia and CKD is needed in the management of a patient with increasing survival as the finding in this study showed shorter time survival in patients with tachycardia and CKD.

Tachycardia at admission and pre-existing CKD could be predictors for worse clinical outcomes in the next six months after discharge. Although these two variables were not statistically significant, which might be related to the insufficient number of respondents, the confidence interval indicated a tendency of higher death rate, as shown in the survival rates on the Kaplan Meier estimate. Higher heart rate during the acute event of HF results from neurohormonal disturbances. This neurohormonal activation acts as a mechanism to compensate for low cardiac output, which is beneficial in the short term [24]. However, in the long term, tachycardia might increase myocardial oxygen demand, induce additional ischemia, and cause arterial stiffness [25]. Most patients in this population had acute decompensation episodes from chronic HF; therefore, they might be previously treated with heart-rate-lowering agents, such as beta-blockers. In this situation, tachycardia at admission may reflect poor heart rate control in chronic HF. Eventually, worsened clinical outcomes in patients with AHF with tachycardia may result from the interaction between neurohormonal alteration, poor heart rate control, suboptimal previous treatment, and severity of the syndrome itself [25-28].

Several established studies have shown that CKD is associated with poor prognosis in both acute and chronic HF. The higher the blood urea and creatinine levels, the worse outcomes in patients admitted with AHF [29-33]. Renal worsening in patients with AHF also contributed to the need for ICU, respiratory failure requiring mechanical ventilation, and prolonged length of stay in the hospital [32]. Notably, CKD presents in >40% of patients with HF [34]. CKD affects HF and vice versa, namely, cardiorenal syndrome to describe the relationship of these two [33]. The risk of cardiac impairment increases gradually with the decline in kidney function. In the long term, CKD causes fluid retention that further increases preload. Resistant hypertension, which is commonly encountered in advanced CKD, could increase the cardiac workload as a consequence of the elevated afterload [30]. In contrast, low cardiac output and congestion as the results of HF could cause hypoperfusion and renal vein congestion, leading to the deterioration of renal function [34]. Even though RAAS acts as a compensatory mechanism in HF, its benefits decline over time, even contributing to further cardiac remodeling which amplifies the clinical progression of HF. Long-term cardiac effects of RAAS are cardiac hypertrophy and fibrosis. Whereas the detrimental effects in the kidney are reduced medullary blood flow, tubular fibrosis, and efferent and afferent arterial vasoconstriction [30,32]. The result of this complex interaction may explain the worse outcome reported in patients with AHF with CKD compared to patients with AHF without pre-existing renal dysfunction.

This study has several limitations. First, the number of subjects was rather small. This unfavorable situation was related to a significant decrease in the number of patients visiting hospitals as the COVID-19 pandemic began in March 2020 in our region. Besides, in the same year, there was a cooperation withdrawal of the national insurance with our institution. This situation further limited the patient’s preference to seek medical service to our center. Second, this evaluation came from a single-center experience and might not represent the characteristics and outcomes of HF in Indonesia’s general population. Third, some parameters from the echocardiography examination were incompletely recorded. Fourth, natriuretic peptide and iron studies from the laboratory were only performed in a small number of patients, precluding further elaboration of these data. Lastly, our six-month follow-up did not include the type of medication with their doses. This situation contributed to the difficulty in evaluating whether the patients had received optimal medication after discharge. Hopefully, an upcoming larger representative nationwide registry could provide additional and valuable information regarding the clinical outcomes of the patients recently hospitalized with AHF.

## Conclusions

This single-center and prospective study analyzed the six-month clinical outcomes following hospital discharge in patients with AHF who had predominantly HFrEF, were overweight or obese, had CAD and hypertension, and presented with ADHF. Poor patient compliance was considered the most common precipitating factor for rehospitalization. Although in-hospital mortality was relatively low, the six-month death and readmission rates were still high and worrisome. Tachycardia on admission and pre-existing CKD had been shown as potential predictors for the worse long-term adverse events in this population.

## Appendices

Contribution details

Leonardo Paskah Suciadi: conceptualization, methodology, validation, investigation, resources, writing-original draft, writing-review and editing, supervision, and project administration

Kevin Wibawa: formal analysis, investigation, writing-original draft, writing-review and editing, and visualization

Giovanni Jessica: formal analysis, investigation, writing-original draft, and visualization

Joshua Henrina Sundjaja: formal analysis, investigation, and writing-review and editing

Irvan Cahyadi: formal analysis and investigation

Bryany Titi Santi: formal analysis and writing-review and editing

Titus Kurnia Hariadi: resources

Firman Tedjasukmana: resources

Nathania Marliani Kristanti: resources

Elisa Feriyanti Pakpahan: resources

Reynold Agustinus Manullang: resources

Antono Sutandar: resources and writing-review and editing
